# Mechanism of action of a T cell-dependent bispecific antibody as a breakthrough immunotherapy against refractory colorectal cancer with an oncogenic mutation

**DOI:** 10.1007/s00262-020-02667-9

**Published:** 2020-07-14

**Authors:** Daisuke Kamakura, Ryutaro Asano, Hiroki Kawai, Masahiro Yasunaga

**Affiliations:** 1 Division of Developmental Therapeutics, Exploratory Oncology Research and Clinical Trial Center, National Cancer Center, 6-5-1 Kashiwanoha, Kashiwa, Chiba 277-8577 Japan; 2 Department of Integrated Biosciences, Graduate School of Frontier Sciences, The University of Tokyo, Chiba, 277-8562 Japan; 3 Department of Biotechnology and Life Science, Graduate School of Engineering, Tokyo University of Agriculture and Technology, Tokyo, 184-8588 Japan; 4 Research and Development Department, LPIXEL Inc., Tokyo, 100-0004 Japan

**Keywords:** Antibody therapeutics, Bispecific antibody (BsAb), T cell-dependent bispecific antibody (TDB), Immunological synapse, Immunotherapy, Colorectal cancer

## Abstract

T cell-dependent bispecific antibody (TDB)-induced T cell activation, which can eliminate tumor cells independent of MHC engagement, is expected to be a novel breakthrough immunotherapy against refractory cancer. However, the mechanism of action of TDBs has not been fully elucidated thus far. We focused on TDB-induced T cell–tumor cell contact as an important initial step in direct T cell-mediated tumor cell killing via transport of cytotoxic cell proteases (e.g., granzymes) with or without immunological synapse formation. Using an anti-EGFR/CD3 TDB, hEx3, we visualized and quantified T cell–tumor cell contact and demonstrated a correlation between the degree of cell contact and TDB efficacy. We also found that cytokines, including interferon-gamma (IFNγ) and tumor necrosis factor-alpha (TNFα) secreted by activated T cells, damaged tumor cells in a cell contact-independent manner. Moreover, therapeutic experiences clearly indicated that hEx3, unlike conventional anti-EGFR antibodies, was effective against colorectal cancer (CRC) cells with mutant KRAS, BRAF, or PIK3CA. In a pharmacokinetic analysis, T cells spread gradually in accordance with the hEx3 distribution within tumor tissue. Accordingly, we propose that TDBs should have four action steps: 1st, passive targeting via size-dependent tumor accumulation; 2nd, active targeting via specific binding to tumor cells; 3rd, T cell redirection toward tumor cells; and 4th, TDB-induced cell contact-dependent (direct) or -independent (indirect) tumor cell killing. Finally, our TDB hEx3 may be a promising reagent against refractory CRC with an oncogenic mutation associated with a poor prognosis.

**Electronic supplementary material:**

The online version of this article (10.1007/s00262-020-02667-9) contains supplementary material, which is available to authorized users.

## Introduction

**Fig. 1:**
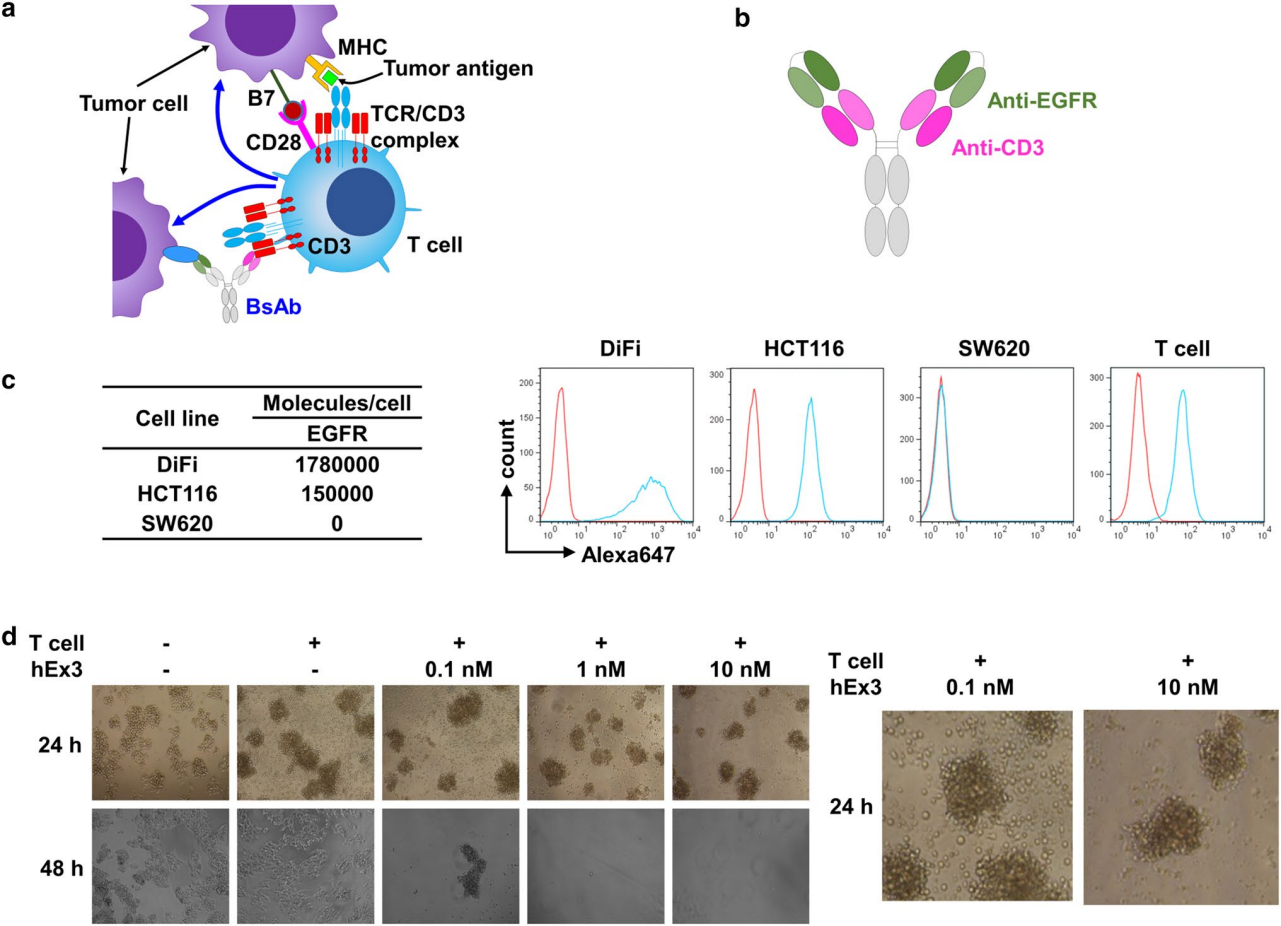
Characteristics of hEx3 (an anti-EGFR/CD3 BsAb). **a** Principle of TDB action. TDB forms a bridge between tumor cells and T cells, inducing T cell activation and subsequent tumor cell killing without recognizing tumor antigen presentation by MHC molecules. **b** Schematic illustration of the hEx3 structure. **c** Binding affinity of hEx3 for CRC cells and T cells, as analyzed by flow cytometry. **d** In vitro study of T cell redirection and tumor cell elimination by hEx3

Colorectal cancer (CRC) is the third most common malignancy worldwide. Despite the fact that mortality rates have been decreasing over recent years because of developments in related knowledge and technologies, the 5-year relative survival rate is still less than 70%, even in advanced countries [1, 2]. Anti-epidermal growth factor receptor (EGFR) monoclonal antibodies (mAbs), such as cetuximab and panitumumab, have been widely used in the treatment of metastatic or advanced CRC [3, 4]. Although these antibodies can kill CRC cells via a neutralizing effect on EGFR signaling, primary or acquired oncogenic mutations in downstream molecules of the EGFR signaling pathway, e.g., KRAS, BRAF, or PIK3CA, can cause therapeutic resistance to anti-EGFR mAbs [5–8]. On the other hand, bispecific antibodies (BsAbs) as next-generation antibody therapies are anticipated to be effective against therapy-resistant or recurrent CRC due to their unique bioactivities not found in conventional therapeutic antibodies. Among BsAbs, we focused on T cell-dependent BsAbs (TDBs), in which an antitumor antigen antibody and an anti-CD3 antibody are combined in one antibody structure. TDBs form a bridge between tumor cells and T cells, which can induce T cell activation. Subsequently, activated T cells can kill tumor cells effectively without T cell receptor (TCR)-mediated recognition of tumor antigen presented by major histocompatibility complex (MHC) molecules (Fig. 1a). Unfortunately, MHC molecules in tumor cells disappear, and disturbed tumor antigen processing and presentation should cause therapeutic resistance or recurrence [9]. On the other hand, TDBs with MHC-peptide-independent activation through TCRs cannot be influenced by these resistance mechanisms [10].

Immunological synapse (IS) formation, as a cutting-edge mechanism for T cell-mediated killing of tumor cells via TCR signaling, is also considered an important mechanism of action (MOA) of TDBs. However, T cells can kill tumor cells without IS formation [11, 12]. In this study, we focused on T cell–tumor cell contact as an important initial step in T cell–mediated tumor cell killing, with or without IS formation. We visualized TDB-induced cell contact to investigate the MOA of our TDB, the anti-EGFR/CD3 BsAb hEx3 [13–15]. We found that hEx3-induced T cell activation was able to cause both cell contact-dependent tumor cell killing (direct tumor cell killing) via cytotoxic cell proteases, e.g., granzymes, and cell contact-independent tumor cell killing (indirect tumor cell killing) via released cytotoxic cytokines, e.g., interferon-gamma (IFNγ) and tumor necrosis factor-alpha (TNFα).

Furthermore, we established CRC cells with a BRAF mutation using a parental DiFi CRC cell line with no BRAF mutations to investigate whether the occurrence of this BRAF mutation affects the efficacy of the TDB hEx3. Unlike a conventional anti-EGFR mAb, hEx3 showed a strong cytotoxic effect regardless of BRAF mutation. In our in vivo study, we also found that hEx3 was delivered to the whole tumor, and accordingly, T cells were redirected to CRC cells, resulting in tumor shrinkage.

Thus, hEx3 is a TDB with a unique MOA and pharmacokinetic and pharmacodynamic profiles and may be a novel, promising alternative to current treatments for refractory CRC.

## Material and methods

### Cells, antibodies, and reagents

Human CRC cell lines (HCT116, SW480, SW620, and HT-29) were purchased from ATCC, and the human CRC cell line DiFi was kindly provided by Dr. Kimio Yonesaka of the Kindai University Faculty of Medicine. DiFi cell lines with or without mutant BRAF were established by transfecting the plasmid pCMV6-BRAF (V600E) or the corresponding empty vector (Origene, Rockville, MD) into parental cells. All CRC cell lines were maintained in Dulbecco’s modified Eagle’s medium (FUJIFILM Wako Pure Chemical, Osaka, Japan) supplemented with FBS (Thermo Fisher Scientific, Waltham, MA) and a 1% penicillin–streptomycin–amphotericin B suspension (FUJIFILM Wako Pure Chemical). Human peripheral blood mononuclear cells (PBMCs) were purchased from Cellular Technology Limited (Cleveland, OH). Human T cells were isolated and expanded by culturing PBMCs with 100 IU/ml recombinant human interleukin-2 (Shionogi, Osaka, Japan) and 10 μg/ml anti-CD3 mAb (OKT3, Tonbo Biosciences, San Diego, CA). These blood cells were cultured in Roswell Park Memorial Institute medium-1640 (FUJIFILM Wako Pure Chemical) supplemented with FBS and a 1% penicillin–streptomycin–amphotericin B suspension.

### Generation of hEx3

We used hEx3-scDb-Fc(H237Y)-HL (referred to as hEx3 simply in this study), a stabilized version of the human Fc-fused hEx3 bispecific diabody with hinge modifications. Highly purified hEx3 molecules were kindly provided by CMIC JSR Biologics Co., Ltd. (Shizuoka, Japan). Briefly, hEx3 molecules were purified through Protein A chromatography followed by ion exchange chromatography.

### Flow cytometry

For analysis of the cell-binding activities of hEx3, flow cytometry was performed. Each set of target cells (DiFi, HCT116, SW620, or isolated T cells) was harvested and incubated with hEx3 (1 μg/ml) in DPBS (Thermo Fisher Scientific) containing 0.1% BSA and 2 mM EDTA (B.E. PBS) for 30 min. After washing with B.E. PBS, an Alexa Fluor 647-conjugated anti-human IgG antibody (Thermo Fisher Scientific) in B.E. PBS was added to the sample as a secondary antibody. Again, after washing with B.E. PBS, 1 μg/ml propidium iodide (Thermo Fisher Scientific) in B.E. PBS was added to stain dead cells. Stained cells were analyzed using a Guava EasyCyte (Melk Millipore, Burlington, MA) with the FlowJo program (Tree Star, Ashland, OR).

### Immunofluorescence staining

The following primary antibodies were acquired from the indicated suppliers: anti-EGFR (polyclonal; R&D Systems, Minneapolis, MN, USA), anti-CD3 (4B10; GeneTex, Irvine, CA, USA), anti-granzyme B (23H8L20; Thermo Fisher Scientific), and anti-IFNγ (polyclonal; Abcam, Cambridge, UK). Secondary antibodies were Alexa Fluor 488-conjugated anti-rabbit IgG (Thermo Fisher Scientific), Alexa Fluor 555-conjugated anti-mouse IgG (Thermo Fisher Scientific), Alexa Fluor 647-conjugated anti-goat IgG (Thermo Fisher Scientific), Alexa Fluor 647-conjugated anti-human IgG (Jackson ImmunoResearch, West Grove, PA), and brilliant violet-conjugated anti-goat IgG (Jackson ImmunoResearch) antibodies were purchased and used as secondary antibodies. In vitro fluorescence imaging was conducted by coculturing DiFi cells with mutant BRAF and isolated T cell lymphocytes in Falcon CultureSlides (Corning, New York, NY), followed by the addition of hEx3. After fixation with 4% paraformaldehyde (PFA) in PBS, each molecule and antibody were stained with the appropriate primary antibody and secondary antibody, respectively.

For an ex vivo study, tumor tissue samples were removed from xenografted mice, which were injected with human T cells, at 8, 24, or 72 h after administration of hEx3, embedded in Tissue Tec optimal cutting temperature compound (Sakura Finetek Japan, Tokyo, Japan) and frozen at − 80 °C until use. Then, the frozen sections were fixed, blocked, and stained with each antibody. The nuclei were stained with DAPI (Thermo Fisher Scientific). Images were obtained using an FV3000 microscope (Olympus, Tokyo, Japan), VS120 Virtual Slide System (Olympus), or BZ-X710 (Keyence, Osaka, Japan).

### Image analysis

To analyze TDB-induced T cell–tumor cell contact, the ratio of the CD3-stained area overlapping with the EGFR-stained area to the total CD3-stained area was calculated. In brief, noise was reduced by Gaussian filtering, and then binary images were created by thresholding. Additionally, EGFR-stained images were processed by dilation operation to connect separated cell surface areas. Finally, pixel numbers for the CD3-stained area and CD3 area overlapping with the EGFR-stained area from multiple images (at least nine images for each group) were summed, and the ratio was calculated. These processes were calculated with python (v3.6.9) and opencv (v4.0.0).

### Assessment of cell cytotoxicity and cytokine secretion

Two anti-EGFR mAbs, namely, cetuximab and panitumumab, were purchased from Merck and Takeda Pharmaceutical, respectively. To evaluate the cytotoxicity of anti-EGFR mAbs, CRC cells were seeded at 5000 cells per well in 96-well plates (Corning) and treated with each mAb for 72 h at 37 °C. For assessment of the T cell-dependent cytotoxicity of hEx3, CRC cells were seeded as described above, except for a change in the number of cells (10,000). The indicated concentrations of hEx3 and isolated T cells were added at an E:T ratio of 10:1 and incubated for 24 h at 37 °C. The percentage of tumor cell killing was determined by counting viable cells with Cell Counting Kit-8 (Dojindo Laboratories, Kumamoto, Japan).

For quantification of the IFNγ and TNFα released from TDB-activated T cells, DiFi and T cells were coincubated at an E:T ratio of 5:1 in 96-well plates, followed by the addition of various concentrations of hEx3. After coculturing for 24 h, the cell supernatants were collected, and the secretion of IFNγ and TNFα was assessed with the Human IFN-gamma ELISA Kit (Proteintech Group, Rosemont, IL) and Human TNF-alpha ELISA Kit (Proteintech Group) according to the manufacturer’s protocol, respectively.

### Analysis of a cell contact-independent tumor cell killing

Indirect T cell activity was analyzed by measuring the cell killing efficacy of conditioned medium from a DiFi/T cell coculture treated with hEx3. DiFi cells were seeded at 10,000 cells per well in a 96-well plate (Corning) and treated with isolated T cells (E:T ratio of 5:1) and hEx3 at the indicated concentrations. After incubating for 24 h at 37 °C, the conditioned medium was harvested by centrifugation and added to independently prepared DiFi cells (10,000 cells per well in a 96-well plate). The percentage of cell killing after 24 h was determined with Cell Counting Kit-8 (Dojindo Laboratories). Next, direct cytotoxicity and indirect cytotoxicity with 100 ng/ml hEx3 were compared at different E:T ratios (5:1, 1:1, or 1:5). To determine the contribution of IFNγ and TNFα to indirect tumor cell killing, conditioned medium harvested from centrifuged DiFi/T cell coculture treated with 100 ng/ml hEx3 was heated at 90 °C for 30 min. The cytotoxicity of heated supernatant with or without 1 ng/ml recombinant IFNγ (PeproTech, Rocky Hill, NJ, USA), 1 ng/ml recombinant TNFα (PeproTech), or the combination of both cytokines was examined as described above.

### hEx3-mediated antitumor effect on tumor/T cell-xenografted mouse models

All animal studies were performed in compliance with the Guidelines for the Care and Use of Experimental Animals established by the Committees for Animal Experimentation of the National Cancer Center. These guidelines meet the ethical standards required by law and comply with the guidelines for the use of experimental animals in Japan.

Female NOD-SCID mice (4 weeks old) were purchased from Charles River Laboratories Japan (Kanagawa, Japan). Mice were subcutaneously inoculated with 1 × 10^7^ DiFi cells with mutant BRAF. When the mean tumor volume reached approximately 150 mm^3^, the mice were randomly divided into groups. Then, selectively isolated T cells were intraperitoneally injected into the mice (4 × 10^7^ cells per mouse). One and 6 days after T cell injection, 1 or 5 mg/kg hEx3 or vehicle was intravenously administered. Tumor volume was measured with a digital caliper every 4 or 3 days and calculated according to the formula: (tumor volume = (length) × (width)^2^ × 1/2). The body weight of the mice was measured at the same time. For the evaluation of toxicity, lung, liver, and spleen were excised 1 week after the administration of 1 mg/kg hEx3 or saline as control. Both lung and liver were fixed with formalin and embedded in paraffin, and each section was stained with hematoxylin and eosin. Spleens were weighed and imaged.

### Statistical analysis

Data with error bars are presented as the mean ± SD. For image analysis of TDB-induced T cell–tumor cell contact, images acquired in a single experiment were randomly divided into three groups in each condition and compared using Student's *t*-test. Statistically significant differences among groups were determined by one-way ANOVA with Tukey analysis or Dunnett analysis for evaluation of cytotoxicity and antitumor efficacy. Student’s t-test was applied to analyze statistical differences in spleen and body weight. All analyses were carried out using R software version 3.6.2.

## Results

### Preparation and characterization of the TDB hEx3

We used the anti-EGFR/CD3 BsAb hEx3, which has two single-chain diabody molecules with an Fc portion that was described previously (Fig. 1b) [15]. First, we conducted flow cytometry analysis to confirm the reactivity of hEx3 to both EGFR and CD3 on CRC cells and T cells, respectively. hEx3 was able to bind EGFR-positive CRC cells in proportion to the number of EGFR molecules but not EGFR-negative cells (Fig. 1c). It was also able to bind CD3-positive T cells specifically.

We then evaluated the redirection of T cells and the cytotoxic effect on DiFi CRC cells in an in vitro hEx3 treatment study. T cells were able to attach to CRC cells, and the number was increased in a dose-dependent manner at 24 h after treatment. Finally, CRC cells were eliminated efficiently after 48 h of treatment according to the number of surrounding T cells (Fig. 1d). Some residual CRC cells showed piecemeal death.

### Visualization and quantification of TDB-induced T cell–tumor cell contact

**Fig. 2:**
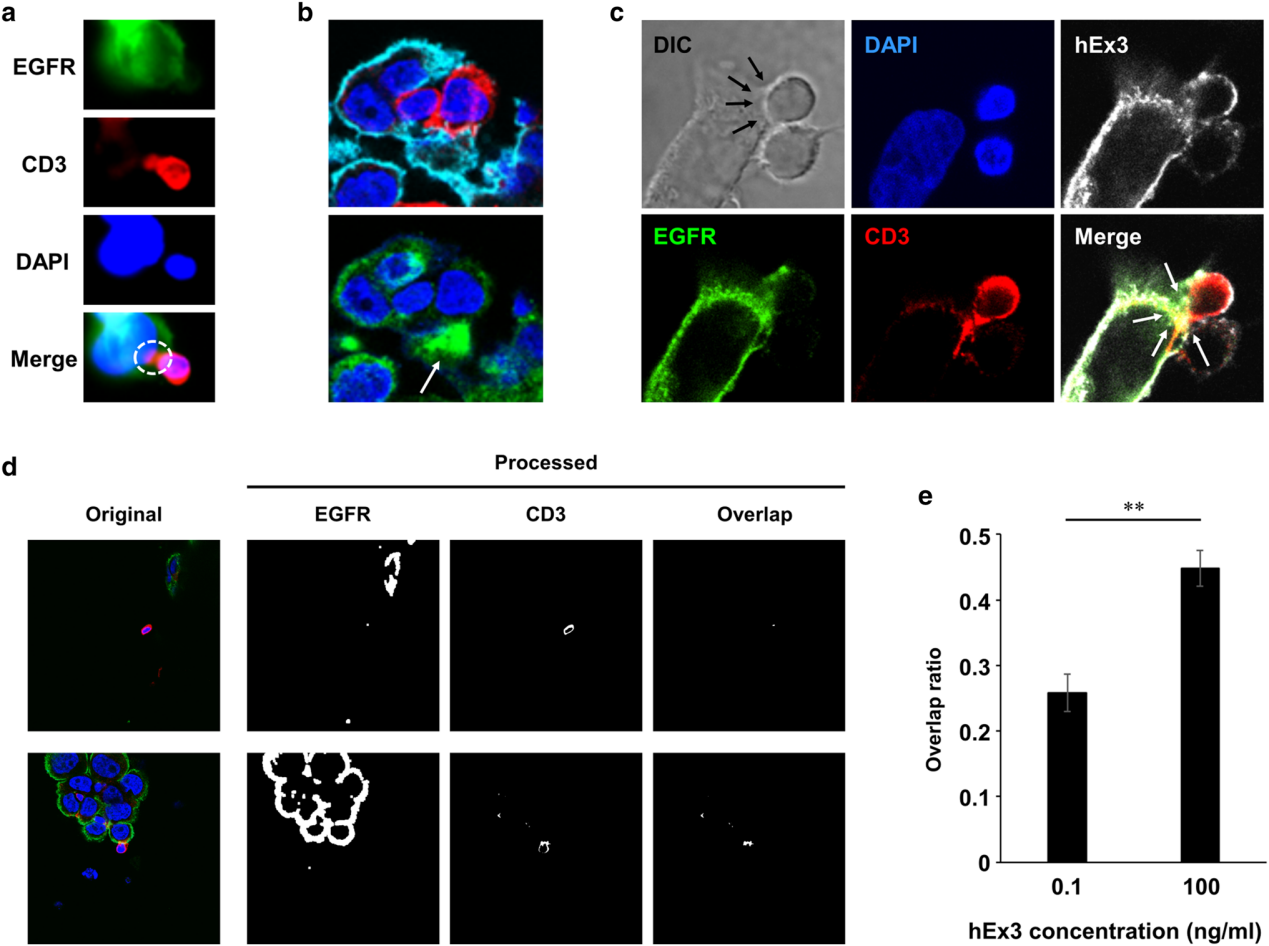
Visualization of TDB-induced T cell–tumor cell contact. **a** Staining of an hEx3-treated tumor/T cell coculture model with DAPI (nucleus, blue) and for EGFR (green), and CD3 (red). The color-overlapping region in the merged image indicated TDB-induced T cell–tumor cell contact as an initial step in T cell-mediated tumor cell killing (white dotted ellipse). **b** Accumulation of granzymes (green) in a damaged DiFi cell bound to a T cell (white arrow). DAPI (nucleus, blue), EGFR (cyan), and CD3 (red). **c** hEx3 binding to both DiFi cells and T cells. The arrows indicate TDB-induced cell contact lesion. **d** Comparison of the efficiency of TDB-induced cell contact between two hEx3 dose groups, the low-dose group (0.1 ng/ml, top) and the high-dose group (100 ng/ml, bottom). EGFR-stained images were processed by a dilation operation during which the pixel number was summed. **e** TDB-induced cell contact ratios calculated with the following formula (total number of overlapped pixels/total number of pixels in processed CD3-stained images). Error bars represent the mean ± SD (*n* ≥ 9). **P < 0.01

Here, we examined TDB-induced T cell–tumor cell contact as an important initial step in T cell-mediated tumor cell killing directly. By staining for both EGFR and CD3, we could clearly visualize cell–cell contact (Fig. 2a). To cause cell damage, transport of cytotoxic granzymes from T cells to tumor cells is needed. We then confirmed strong accumulation of granzyme in damaged CRC cells bound by T cells (Fig. 2b). In these assays, we also found that hEx3 bound both EGFR on CRC cells and CD3 on T cells (Fig. 2c). Additionally, overlapping signals caused by EGFR and CD3 in the boundary site between CRC cells and T cells were considered to represent active binding sites of TDB. Accordingly, to determine the efficacy of hEx3, we conducted a quantitative analysis of a TDB-induced T cell–tumor cell contact. In an imaging analysis, the cell contact ratio was calculated based on the pixel numbers of the CD3-stained spots overlapping with the EGFR-stained spots in a given area (Fig. 2d). The cell contact ratio in the high-dose (100 ng/ml) hEx3 treatment group was significantly higher than that in the low-dose (0.1 ng/ml) treatment group (Fig. 2e). Collectively, these results indicate that hEx3 can redirect T cells toward CRC cells and cause direct cell damage.

### Cell contact-independent tumor cell killing caused by TDB treatment

**Fig. 3:**
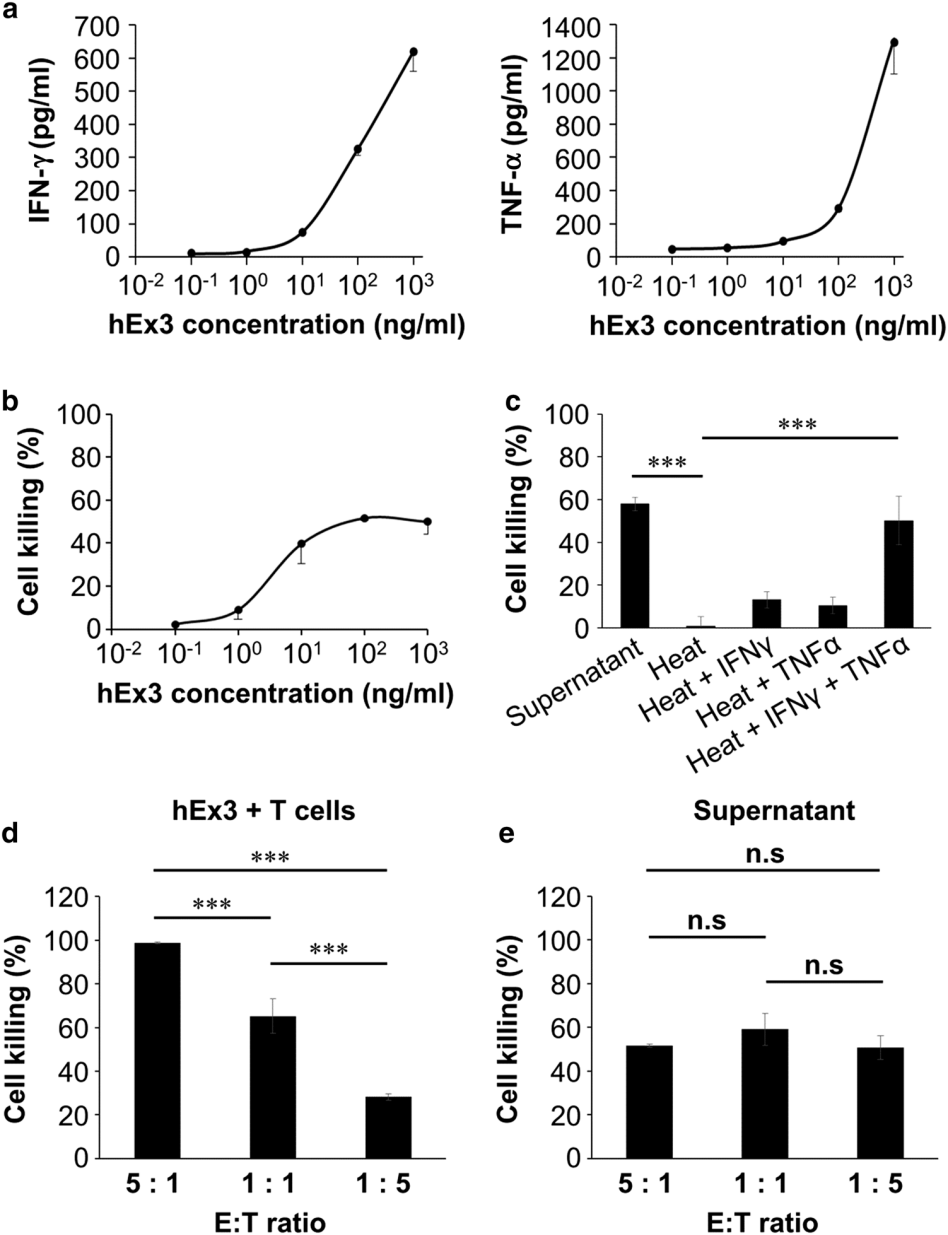
TDB-induced cell contact-independent tumor cell killing. **a** Assessment of the secretion of IFNγ (left) and TNFα (right) from T cells driven by hEx3 (E:T ratio of 5:1). Error bars represent the mean ± SD (*n* = 3). **b** Indirect cytotoxicity of cell culture supernatants from a coculture of activated T cells and DiFi cells (E:T ratio of 5:1) treated with hEx3. Error bars represent the mean ± SD (*n* = 3). **c** Indirect cytotoxicity of heat-inactivated supernatant (Heat), addition of 1 ng/ml recombinant IFNγ (Heat + IFNγ), 1 ng/ml recombinant TNFα (Heat + TNFα), or both in combination (Heat + IFNγ + TNFα). Pre–heat-inactivated supernatant (Supernatant) was used as control. Error bars represent means ± SD (*n* = 3). ***P < 0.001. **d**, **e** The efficacy of tumor cell killing by hEx3-induced T cells (**d**) or by cell culture supernatants (**e**) at different E:T ratios. The number of DiFi cells was fixed at 10,000 cells per culture. hEx3 (100 ng/ml) was added to each culture. Error bars represent the mean ± SD (*n* = 3). ***P < 0.001, *n.s.* not significant

T cell–tumor cell contact as an initial step in T cell-mediated tumor cell killing is obviously important for cell damage of hEx3. However, the cytotoxic cytokine IFNγ and TNFα from T cells are also an important indicators of T cell activation. We then examined whether hEx3 enhances the secretion of IFNγ and TNFα from T cells. ELISA data showed that both IFNγ and TNFα were released from activated T cells in response to hEx3 treatment in a dose-dependent manner, although cell contact-dependent tumor cell killing is the major MOA of the TDB (Fig. 3a).

We speculated that the released IFNγ and TNFα could also mediate killing of tumor cells in a cell contact-independent manner. Actually, the supernatant from a coculture of CRC cells with T cells activated via hEx3 caused cell damage in an hEx3-dose-dependent manner (Fig. 3b). Subsequently, we conducted a heat inactivation assay to identify the molecules contributing to cell contact-independent tumor cell killing. The heat-inactivated supernatant had no cytotoxic activity. On the other hand, the heat-inactivated supernatant with either recombinant IFNγ or recombinant TNFα exhibited partial restoration of tumor cell killing activity. Moreover, the tumor cell killing activity of the heat-inactivated supernatant was substantially restored by the addition of IFNγ and TNFα (Fig. 3c).

Generally, an effector-to-target (E:T) ratio of 5:1 is used for the evaluation of TDBs. However, in clinical tumor samples, there tends to be a small number of T cells compared with the large number of tumor cells. Therefore, we evaluated both cell contact-dependent and -independent tumor cell killing at different E:T ratios including 5:1, 1:1, and 1:5. Interestingly, the efficacy of cell contact-dependent tumor cell killing was decreased considerably when the E:T ratio was decreased (Fig. 3d). On the other hand, the efficacy of the coculture supernatant representing cell contact-independent tumor cell killing was not changed between the E:T ratios of 5:1, 1:1, and 1:5 (Fig. 3e). Overall, our data suggest that hEx3 has two types of MOAs, cell contact-dependent (direct) and -independent (indirect). The latter would be effective even against tumors with low T cell infiltration.

### The TDB hEx3 is effective against KRAS-, BRAF-, or PIK3CA-mutant CRC cells resistant to anti-EGFR mAbs

**Fig. 4:**
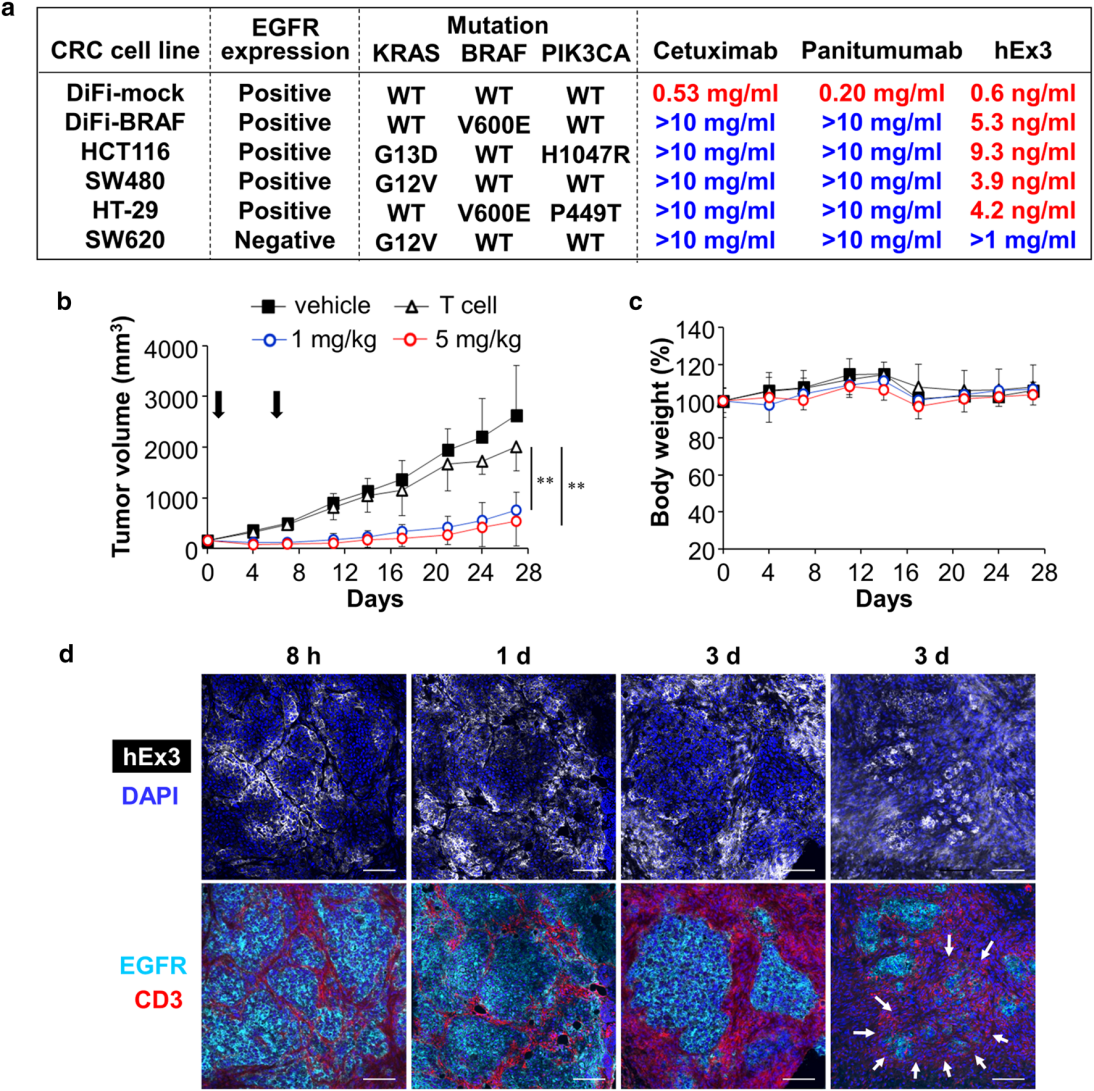
T cell-dependent antitumor efficacy of hEx3 against tumor/T cell-xenografted mice. **a** In vitro cytocidal efficacies of cetuximab, panitumumab, and hEx3 against EGFR-positive CRC cell lines (DiFi-mock, DiFi-BRAF, HCT116, SW480, and HT-29) and an EGFR-negative CRC cell line (SW620). Each IC_50_ value is indicated. **b** In vivo antitumor effect of hEx3 on a mouse xenograft model in the presence of human T cells. Mice bearing subcutaneous DiFi-BRAF tumors were administered saline (T cell), 1 mg/kg hEx3 (1 mg/kg), or 5 mg/kg hEx3 (5 mg/kg) intravenously on days 1 and 6, following intraperitoneal T cell injection on day 0. The control group was treated with saline (vehicle). Error bars represent the mean ± SD (*n* = 5). **P < 0.01. **c** The % change in body weight in treated mice. Error bars represent the mean ± SD. **d** Ex vivo immunohistochemical analyses at 8 h, 1 day, and 3 days after hEx3 administration with DAPI staining (nucleus, blue) and staining for hEx3 (white), EGFR (cyan), and CD3 (red). The arrows indicate tiny tumor masses representing piecemeal death. Scale bar = 100 µm

KRAS, BRAF, and PIK3CA mutations in CRC are well-known markers that cause therapeutic resistance to anti-EGFR therapy. We established DiFi cells with a BRAF mutation (DiFi-BRAF) by gene transfer into parental DiFi cells with wild-type KRAS, BRAF, and PIK3CA [16, 17]. Additionally, DiFi cells transferred with an empty vector were used as control cells (DiFi-mock). Although DiFi-mock cells were sensitive to anti-EGFR mAb therapy, specifically cetuximab or panitumumab, DiFi-BRAF cells showed resistance to these drugs. In contrast, hEx3 showed strong cytotoxicity against both DiFi-mock and DiFi-BRAF cells (Fig. 4a).

Subsequently, we evaluated the efficacy of anti-EGFR mAbs and hEx3 against other CRC cell lines with mutant KRAS, BRAF, or PIK3CA. Consequently, all cell lines with these mutations showed resistance to the anti-EGFR mAbs, whereas they were sensitive to hEx3. Next, we evaluated the antitumor effect of hEx3 against a DiFi-BRAF xenograft model. hEx3 showed a stronger antitumor effect than control saline or adoptive T cell treatment only (Fig. 4b). Moreover, we confirmed the safety of hEx3; there were no adverse events, including clear body weight loss, lung injury, liver injury, or splenomegaly, during treatment (Fig. 4c, supplementary Fig. 1).

### Distribution of the TDB hEx3 and redirection of T cells within tumor tissue

Preclinical pharmacokinetic studies are very important to predict clinical efficacy. Disturbances in both antibody delivery and T cell infiltration are critical issues in the clinical development of a TDB. Therefore, we examined the distribution of hEx3 and redirection of T cells within tumor tissue. hEx3 was distributed throughout the whole tumor area from 8 h to 3 days after injection (Fig. 4d). In accordance with the hEx3 distribution, T cells spread gradually from pre-administration to 3 days afterward (Supplementary Fig. 2a). Moreover, T cells with hEx3 were consistently present in the boundary site between CRC and T cells from 8 h to 3 days after the injection. By contrast, the abundance of T cells without hEx3 increased over the same time course (Supplementary Fig. 2b). Furthermore, IFNγ-positive T cells were observed in both lower and higher T cell infiltration areas, but they were distant from the boundary site between CRC and T cells (Supplementary Fig. 2c). Thereby, tumor cells were surrounded and eliminated by the T cells. Many tiny tumor masses appeared at 3 days after injection of hEx3, which was recognized as piecemeal death. On the basis of these findings, we considered that most T cells were redirected into the tumor following the active targeting of hEx3 as specific binding to CRC cells and T cells within tumor tissue. In addition, both cell contact-independent (indirect) and -dependent (direct) tumor cell killing may be important for tumor shrinkage in vivo, as observed in the in vitro cytotoxic study.

## Discussion

BsAbs, which have two antigen-binding sites in one IgG structure, are artificially engineered from two different antibodies [18]. There are more than 100 design formats for them [19]. Among them, TDBs are composed of a tumor cell-binding site and an immune cell-binding site. They can redirect T cells through CD3 binding. Although conventional immunotherapy requires TCR-dependent activation via interaction with tumor antigens presented by MHC molecules, the very high polymorphism of human MHC genes makes it difficult to develop common immunotherapies or regulate immune reactions stably and steadily. Moreover, tumor cells can escape T cell attack by downregulating MHC expression, which leads to therapeutic resistance to conventional immunotherapy [20, 21]. By contrast, both T cells redirected by a TDB and chimeric antigen receptor (CAR)-T cells, which are genetically engineered redirected T cells with an antibody-based CAR, can attack tumor cells independent of MHC engagement [10, 22–24].

An immune desert microenvironment and T cell exhaustion are common issues in the clinical application of TDBs and CAR-T cells as well as conventional immunotherapies. Combination of these therapies with immune checkpoint blockade (ICB), such as anti-PD-1, anti-PD-L1, and anti-CTLA-4 mAbs, would be effective to overcome the latter issue. Moreover, genetic engineering, such as overexpression of JUN or TOX in CAR-T cells, could also be useful for escaping the exhausted state [25, 26]. However, regarding the former issue, among immunotherapies, TDBs have advantages because they can redirect circulating T cells into the tumor area. We found that the distribution of the TDB hEx3 led to the gradual migration and distribution of T cells around tumor cells. hEx3 has the properties of both passive targeting as a high-molecular-weight (HMW) agent and active targeting as a specific antibody. TDB technologies allow the targeted and selective delivery of both hEx3 and T cells into the tumor area.

**Fig. 5:**
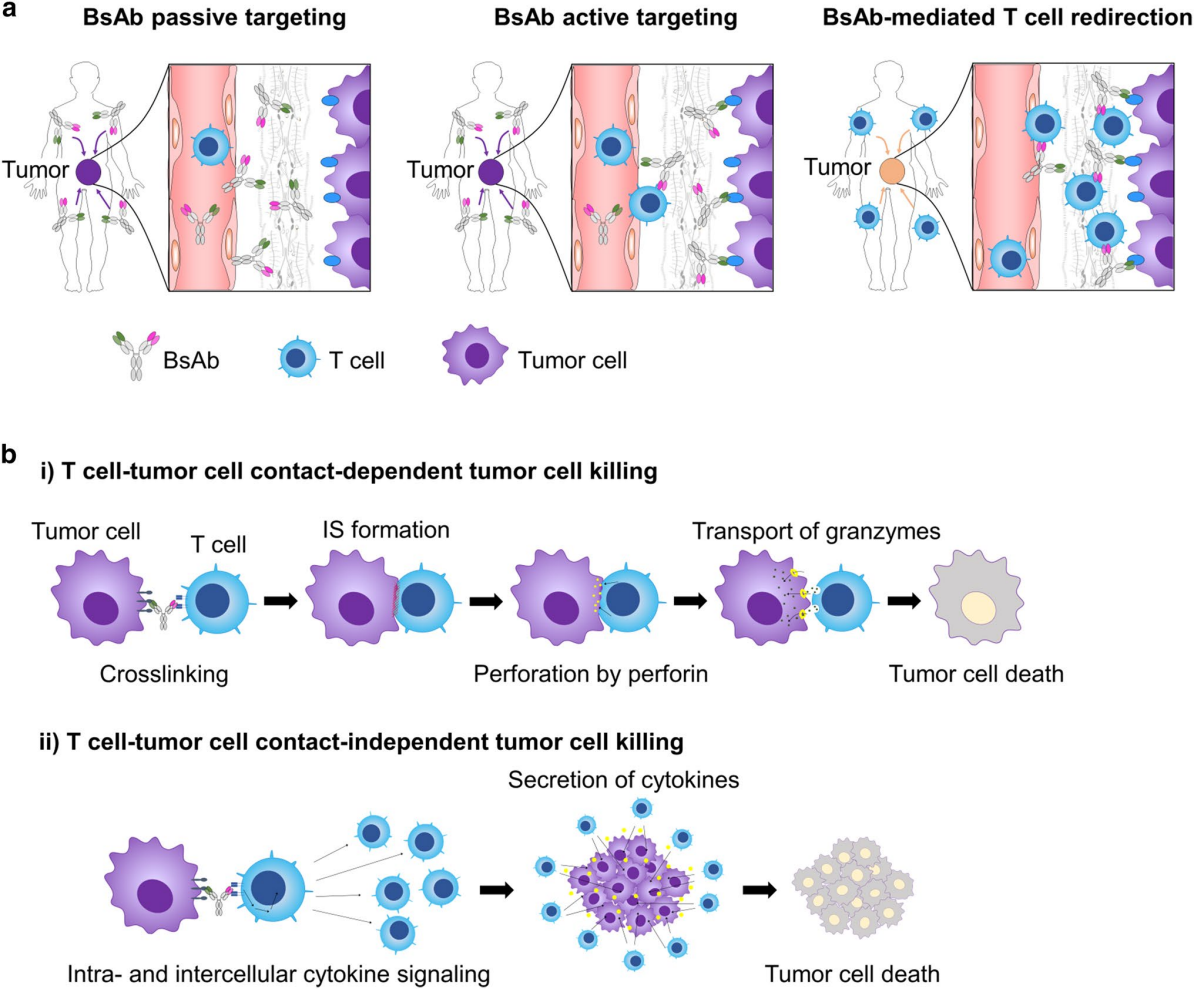
Schematic models depicting the MOAs of the TDB. **a** In the early stage of the MOA, there are three steps: (1) passive targeting of the TDB into the tumor stroma area as a size-dependent property (left), (2) active targeting of the specific TDB by binding to tumor cells (middle), and (3) redirection of T cells by the TDB toward tumor cells (right). **b** In the last step of the MOA, there are two effects. i) In a manner that depends on TDB-induced T cell–tumor cell contact (direct), T cells driven by the TDB directly kill tumor cells through the intracellular perforin–granzyme pathway and induce tumor cell death (top). ii) In a manner that is independent of TDB-induced T cell–tumor cell contact (indirect), the TDB activates T cells and induces tumor cell killing via release of cytotoxic cytokines, including IFNγ and TNFα (bottom)

HMW agents, e.g., liposomes and micelles, can accumulate in a tumor selectively via the enhanced permeability and retention (EPR) effect via passive targeting [27]. Antibodies, including TDBs typically in an IgG format-like hEx3, which has a molecular weight of approximately 150,000 Da and a size of approximately 10 nm, can undergo passive targeting for tumor-selective accumulation over a long period of time. Moreover, TDBs can bind tumor cells specifically through active targeting mediated by recognition of tumor-specific antigens. Furthermore, TDBs can redirect circulating T cells into the tumor area and form a bridge between T cells and tumor cells (Fig. 5a). Recently, we reported the MOA of an antibody–drug conjugate (ADC) as another next-generation antibody therapeutic for drug delivery. Although TDBs and ADCs show unique MOA features, controlled-release payload-mediated tumor cell killing and activated T cell-mediated killing in the last MOA step, both passive targeting and active targeting are very important common steps for the delivery of TDBs and ADCs [28, 29].

The IS is the structure generated at the interface between a T cell and an antigen-presenting cell (APC) or a target cell [30]. Many imaging-based studies have revealed the associations of the IS with antigen recognition, adhesion, and signaling cascades in T cells [31–33]. The importance of the IS for T cell functions has been indicated concerning the area between a T cell and an APC. However, some authors reported that stable IS formation and complete signaling are not required for the cytotoxic effect of T cells [11, 12]. To avoid this complex issue related to IS formation, we focused on TDB-induced T cell–tumor cell contact as an important initial step of T cell-mediated tumor cell killing, with or without IS formation, and established a quantification method using fluorescence microscopy by visualizing the overlap between the tumor antigen EGFR and the T cell surface receptor CD3. Although this simple assay system would be useful to evaluate the effectiveness or efficacy of TDBs simply and could be conducted conveniently in clinical studies, more sophisticated methods to examine IS formation markers, such as PKCθ, CD45, LFA-1, Lck, and ZAP-70, might be necessary to distinguish cell contact-dependent tumor cell killing with IS formation from killing without IS formation [34].

More importantly, we also found two types of MOAs for our TDB, cell contact-dependent (direct) and -independent (indirect) tumor cell killing (Fig. 5b). In an in vitro study, at a high E:T ratio, cell contact-dependent tumor cell killing was effective, whereas at a low E:T ratio, potency was drastically decreased. The latter situation is considered one cause of therapeutic resistance or recurrence after TDB treatment. However, the release of cytotoxic cytokines, including INFγ and TNFα, could compensate for the failure of cell contact-dependent tumor cell killing. From this viewpoint, the activation of T cells within tumor tissue is very important. Unfortunately, TDB-induced T cell activation seems to be weaker than CAR-T cell activation because the former is dependent on CD3 signaling only, but the latter can use many activation signaling pathways simultaneously, e.g., the combination of one or two costimulatory molecules (CD28, 4-1BB, etc.) and the CD3 domain [35]. Therefore, new antibody engineering to enhance T cell activity that equals or exceeds CAR-T cell activity is strongly desired.

EGFR, a receptor tyrosine kinase, is overexpressed in CRC and can activate its downstream signaling pathways that promote the progression of CRC. Therefore, anti-EGFR mAbs that can block signaling pathways, e.g., cetuximab and panitumumab, were developed and are used against advanced or metastatic CRC. Mutation of the KRAS, BRAF or PIK3CA oncogene downstream of EGFR signaling is observed frequently and associated with a poor prognosis in CRC. Moreover, these mutations can enable CRC cells to proliferate and expand in an EGFR-independent manner, resulting in therapeutic resistance against anti-EGFR mAbs. We demonstrated that the TDB hEx3 was able to efficiently kill CRC cells with or without these mutations. Currently, molecular targeted agents (MTAs), including neutralizing antibodies, are widely used in the treatment of cancer. Nevertheless, given the tremendous genetic heterogeneity and mutation acquisition in the patient population, resistance to MTAs emerges rapidly. New MTAs could be developed as mutations or overexpressed proteins arise, but this becomes a costly game of whack-a-mole [36]. TDBs might break this vicious cycle of treatments. Otherwise, the combination of a TDB and an MTA would produce more favorable outcomes than either monotherapy, e.g., the immunological cell death caused by some MTAs might enhance T cell activation within tumor tissue.

We succeeded in visualizing TDB-induced T cell–tumor cell contact as an important initial step in T cell-mediated tumor cell killing, and thus established a convenient quantification method to examine the MOA of a TDB. Therefore, we propose that TDBs have four action steps: 1st, passive targeting into the tumor stromal area as a size-dependent property of the TDB; 2nd, active targeting via antigen-specific binding of the TDB to tumor cells; 3rd, redirection of T cells toward tumor cells; and 4th, two MOAs: TDB-induced cell contact-dependent (direct) or -independent (indirect) tumor cell killing. These double MOAs will be clinically beneficial in the treatment of tumors in which there is a mixed condition of high and low T cell:tumor cell ratios.

Finally, our therapeutic experience clearly indicates that our TDB hEx3, as a unique next-generation therapeutic antibody, may be a promising reagent against refractory CRC with an oncogenic mutation associated with a poor prognosis.

### Electronic supplementary material

Below is the link to the electronic supplementary material.

Supplementary file1 (PDF 1580 kb)

## Data Availability

The source of data and materials are described in the manuscript, in support of the findings.

## Abbreviations

BsAb: Bispecific antibody
CRC: Colorectal cancer
EPR: Enhanced permeability and retention
IS: Immunological synapse
MTA: Molecular targeted agent
TDB: T cell-dependent bispecific antibody
