# Supplementary material for: Mechanism of action of a T cell-dependent bispecific antibody as a breakthrough immunotherapy against refractory colorectal cancer with an oncogenic mutation
Source: Cancer Immunol Immunother. 2020 Jul 14;70(1):177–88. doi: 10.1007/s00262-020-02667-9 (PMC7838078; doi:10.1007/s00262-020-02667-9)
Supplement: Supplementary file 1 — Supplementary file1 (PDF 1580 kb) [file 262_2020_2667_MOESM1_ESM.pdf]

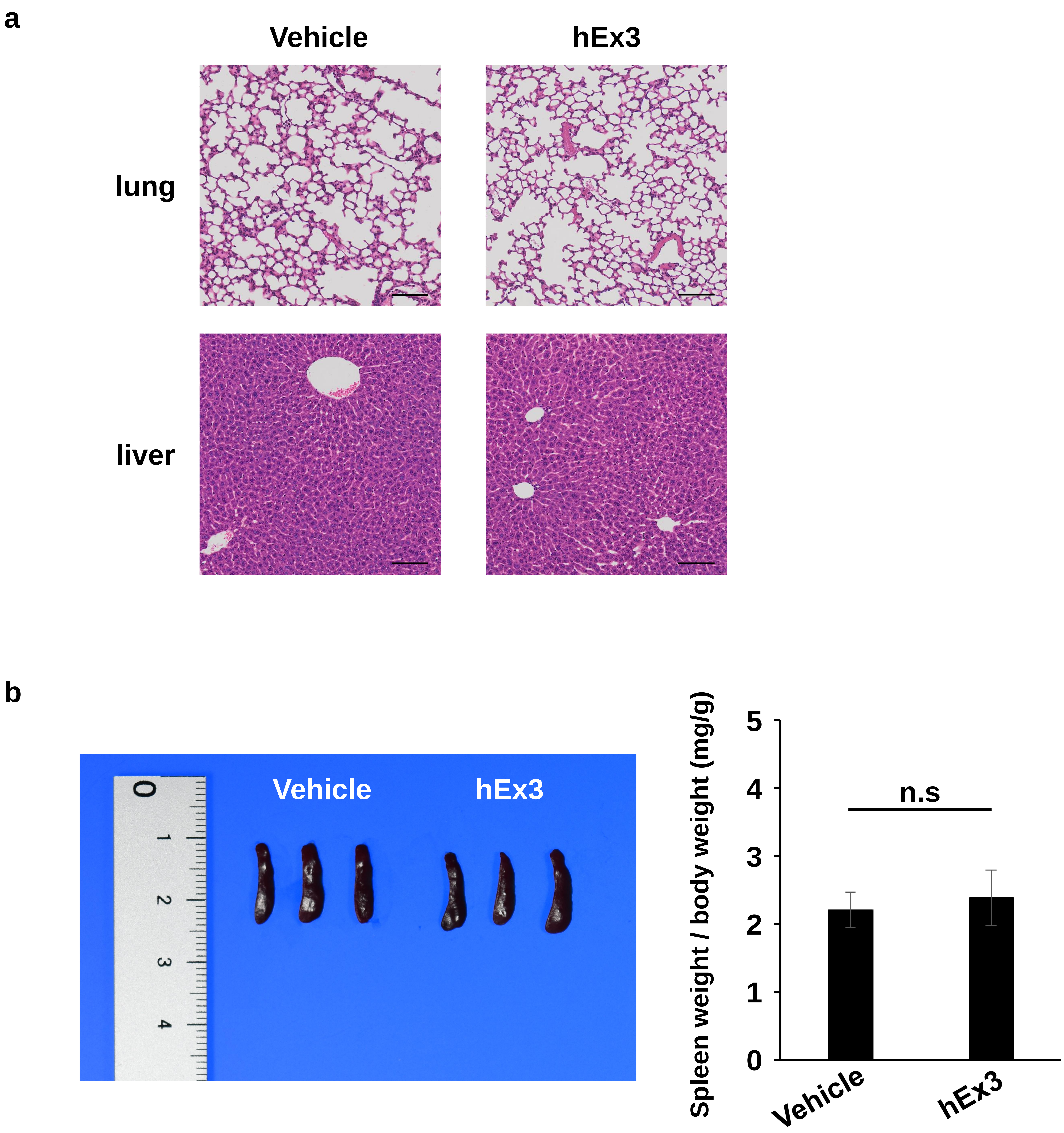

Supplementary Fig. 1 Safety evaluation of hEx3 administration in mice. (a) H&E staining of mouse lung and liver 1 week after injection of hEx3 or saline (vehicle). Scale bar = 100 µm. (b) Size (left) and weight (right) measurements of mouse spleen treated by hEx3 or saline (vehicle) are shown. Each weight value is normalized against individual body weight, as indicated [spleen weight / body weight (mg/g)]. Error bars represent means ± SD (n=3). n.s. = not significant

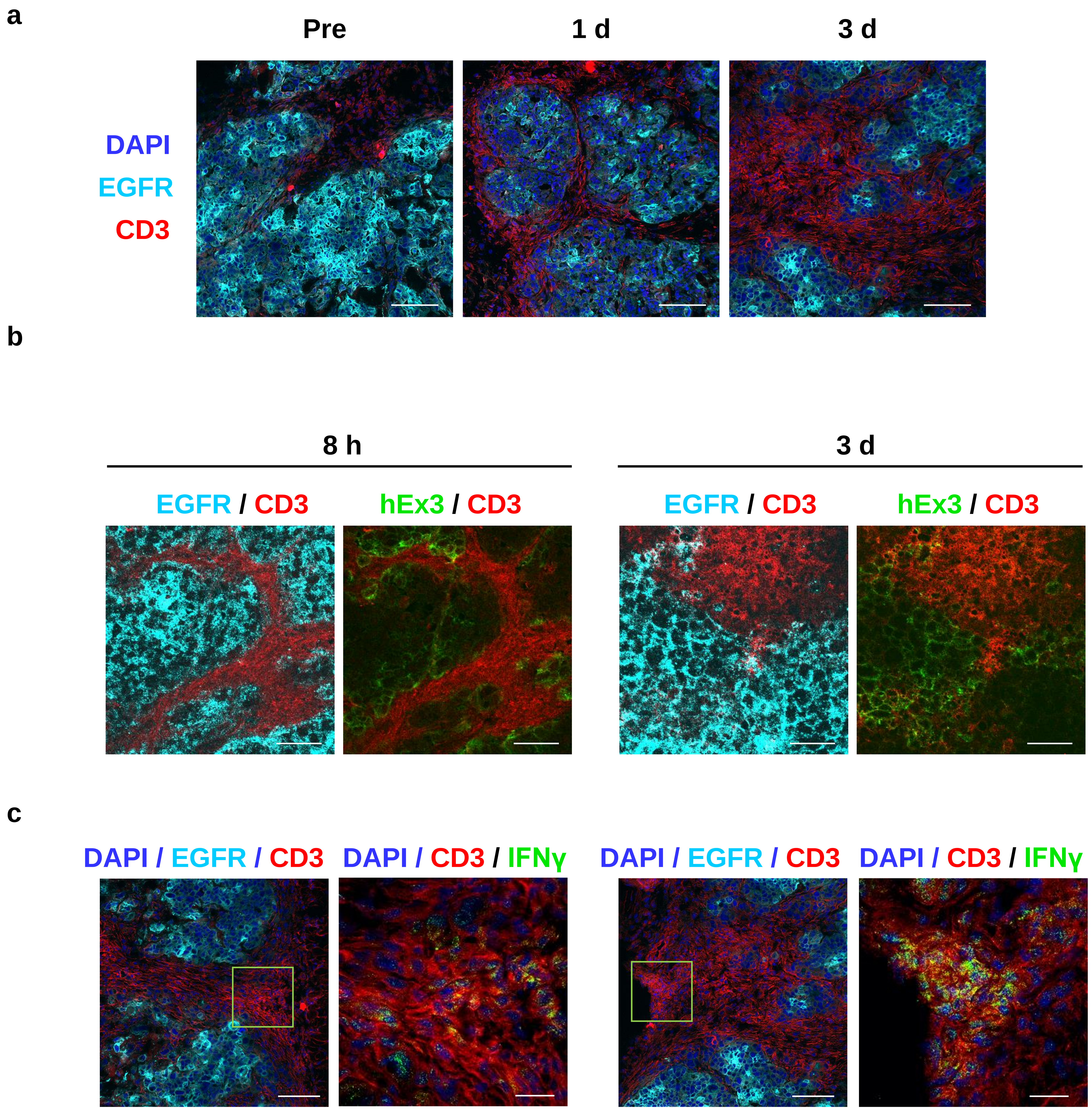

Supplementary Fig. 2 Ex vivo staining of hEx3-treated tumor.
(a) Distribution of CD3-positive T cells (Red) within tumor tissue at pre-administration (Pre) and 1 day (1d) or 3 days (3d) after hEx3 administration. DAPI staining (nucleus) is shown in blue, and EGFR in cyan. Scale bar = 100 µm. (b) Ex vivo immunohistochemical analyses at 8 h and 3 days after hEx3 administration with staining for hEx3 (green), EGFR (cyan), and CD3 (red). Scale bar = 50 µm. (c) Fluorescence staining for IFNγ (green) in tumor tissue with lower– (left) or higher–cell infiltration area (right) 3 days after hEx3 administration. Scale bars = 100 µm (low-power fields in left panels), 25 µm (high-power fields in right panels). Nucleus (blue), EGFR (cyan), CD3 (red)
